# Combinatory Effect of Pequi Oil (*Caryocar brasiliense*)-Based Nanoemulsions Associated to Docetaxel and Anacardic Acid (*Anacardium occidentale*) in Triple-Negative Breast Cancer Cells In Vitro

**DOI:** 10.3390/pharmaceutics16091170

**Published:** 2024-09-05

**Authors:** Alicia Simalie Ombredane, Natália Ornelas Martins, Gabriela Mara Vieira de Souza, Victor Hugo Sousa Araujo, Ísis O. Szlachetka, Sebastião William da Silva, Márcia Cristina Oliveira da Rocha, Andressa Souza de Oliveira, Cleonice Andrade Holanda, Luiz Antonio Soares Romeiro, Elysa Beatriz de Oliveira Damas, Ricardo Bentes Azevedo, Graziella Anselmo Joanitti

**Affiliations:** 1 Laboratory of Bioactive Compounds and Nanobiotechnology (LCBNano), University of Brasilia, Campus Universitário—Centro Metropolitano, Ceilandia Sul, Brasilia 72220-275, Brazil; alicia.ombredane@gmail.com (A.S.O.); nataliaornelas8@gmail.com (N.O.M.); gabimvs@hotmail.com (G.M.V.d.S.); victorhunterhsa@hotmail.com (V.H.S.A.); elysadamas@gmail.com (E.B.d.O.D.); 2 Post-Graduation Program in Nanoscience and Nanobiotechnology, University of Brasilia, Campus Universitário Darcy Ribeiro, Brasilia 70910-900, Brazil; isis_szlachetka@hotmail.com (Í.O.S.); swsilva@unb.br (S.W.d.S.); marcia.cristinaor@gmail.com (M.C.O.d.R.); razevedo@unb.br (R.B.A.); 3 Laboratory of Optical Espectroscopy, Physics Institute, University of Brasilia, Campus Universitário Darcy Ribeiro, Brasilia 70910-900, Brazil; 4 Graduate Program in Pharmaceutical Sciences, Department of Pharmacy, Health Sciences Faculty, University of Brasilia, Brasilia 70910-900, Brazil; andressa901@gmail.com (A.S.d.O.); cleoniceandradeholanda@gmail.com (C.A.H.); luizromeiro@unb.br (L.A.S.R.); 5 Laboratory of Development of Therapeutic Innovations (LDT), Center for Tropical Medicine, Faculty of Medicine, University of Brasilia, Brasilia 70910-900, Brazil

**Keywords:** nanoemulsion, pequi oil, *Caryocar brasiliense*, *Anacardium occidentale*, breast cancer, docetaxel, anacardic acid, combinatory therapy

## Abstract

Combination therapy integrated with nanotechnology offers a promising alternative for breast cancer treatment. The inclusion of pequi oil, anacardic acid (AA), and docetaxel (DTX) in a nanoemulsion can amplify the antitumor effects of each molecule while reducing adverse effects. Therefore, the study aims to develop pequi oil-based nanoemulsions (PeNE) containing DTX (PDTX) or AA (PAA) and to evaluate their cytotoxicity against triple-negative breast cancer cells (4T1) in vitro. The PeNE without and with AA (PAA) and DTX (PDTX) were prepared by sonication and characterized by ZetaSizer^®^ and electronic transmission microscopy. Viability testing and combination index (CI) were determined by MTT and Chou-Talalay methods, respectively. Flow cytometry was employed to investigate the effects of the formulations on cell structures. PeNE, PDTX, and PAA showed hydrodynamic diameter < 200 nm and a polydispersity index (PdI) of 0.3. The association PDTX + PAA induced a greater decrease in cell viability (~70%, *p* < 0.0001) and additive effect (CI < 1). In parallel, an association of the DTX + AA molecules led to antagonism (CI > 1). Additionally, PDTX + PAA induced an expressive morphological change, a major change in lysosome membrane permeation and mitochondria membrane permeation, cell cycle blockage in G2/M, and phosphatidylserine exposure. The study highlights the successful use of pequi oil nanoemulsions as delivery systems for DTX and AA, which enhances their antitumor effects against breast cancer cells. This nanotechnological approach shows significant potential for the treatment of triple-negative breast cancer.

## 1. Introduction

Breast cancer is one of the most common cancer types among women worldwide [1]. Conventional therapies such as chemotherapy are recommended to be employed mainly in the early stages of cancer to increase chances of cure. However, since it is known that different and heterogeneous cancer cell populations can be present in the same tumor mass, some cells may be resistant to the chemotherapeutic approach chosen, thereby challenging treatment success [2]. Triple-negative breast cancer (TNBC) is considered as the most aggressive breast cancer subtype [1,3]. TNBC is characterized by low amounts of or the absence of estrogen, progesterone, and human epidermal growth factor 2 (HER2) receptors’ expression [4]. The high heterogeneity and high level of metastasis of TNBC contributed to limited options for treatment and challenged the development of therapeutic targets [5]. In this context, a combination of therapies represents a great alternative for cancer treatment where different drugs are able to achieve different cellular targets, which increases antitumoral activity and reduces side effects [2,6,7].

Drug combination is one type of combinatory therapy approved by the FDA (Food and Drugs Administration). A chemotherapeutic agent acting on tubulin stabilization, known as Docetaxel (DTX), is commonly used in clinical administration and has shown to be highly efficient for metastatic breast cancers, although it is involved in several side effects. Other drugs such as Pertuzumab, Gemcitabine, Ramucirumab, Pevonedistat, and cis-platin had been successfully associated with DTX in the treatment of breast cancer, prostate cancer, and non-small cell lung cancer [6,7,8,9].

Anacardic acid (AA) is a bioactive compound isolated from cashew nut (*Anacardium occidentale*) and has been described to present antitumor activities by reducing cell proliferation [10,11,12] and inducing apoptosis in cancer cells [13]. Additionally, AA had been shown to be a great ligand for targeted cancer therapy because of its high affinity to vascular endothelial growth factor (VEGF) receptors that are overexpressed in cancer cells [14]. Some studies reported the association of AA and DTX in nanoparticles against breast cancer [7,14]. However, in these cases, AA was used as a surface ligand to interact specifically with cancer cell receptors (e.g., VEGF). In the present study, AA and DTX were used as antitumor agents acting in different cellular targets. To achieve this goal, nanotechnology strategies were chosen to enable their future application in lived organisms since both molecules present a hydrophobic nature. Nanoparticles have been increasingly used as drug delivery systems for cancer treatment and as carriers for hydrophobic compounds [15,16]. These systems increase drug internalization into targeted cells and avoid their early degradation, reducing side effects [17]. Nanoemulsion is one type of nanometric drug delivery system characterized by a mixture of aqueous and oil phases stabilized by surfactants [18,19,20]. Usually, the oil phase has a structural role in forming stable nanoemulsions that are able to carry hydrophobic molecules. Interestingly, in some cases, the oil phase can be chosen according to its biological properties [21,22] and plays a dual role, acting both as structural and antitumor agents in synergism with the carried molecules.

Brazilian Cerrado represents an expressive plant biodiversity environment. Pequi *(Caryocar brasiliense* Camb.) is a native tree of Brazilian Cerrado, rich in vitamin C, carotenoids, and phenolic compounds [23,24,25,26,27]. Pequi oil has demonstrated anticancer activity in animal models. Oral supplementation of this oil led to the inhibition of tumor growth, reduction of side effects induced by chemotherapeutic drugs, and potentiation of anticancer effects of magnetic hyperthermia therapy [28,29,30]. In addition, the design, characterization, stability, and cytotoxic effects of pequi oil-based nanoemulsion (PeNE) on breast cancer cells in vitro have been previously reported [21,22].

Therefore, considering the above-mentioned advantages of therapies associating different antitumor compounds, the aim of this study was to design and characterize PeNE nanoformulations, which separately entrap AA and DTX, and also investigate their combined effects on the cytotoxicity, proliferation, and key cellular structures/organelles of triple-negative breast cancer cells (4T1) in vitro.

## 2. Materials and Methods

### 2.1. Materials

The oil used in this study was extracted from the fruit by cold pressing and filtration and was donated from Farmacotécnica^®^ (a pharmacotechnical development company, Brasilia, Brazil). The characterization of the pequi oil investigated in this study was previously reported by Ombredane and coworkers (2022), also available on the PhD thesis of Ombredane, A.S. (2021) [22,31]. Docetaxel was purchased from Sigma-Aldrich (St. Louis, MO, USA). Anacardic acid was obtained from cashew nut (*Anacardium occidentale*) and characterized according to Sahin and coworkers (2022) [32]. The materials used for cell culture—ethanol, penicillin, streptomycin, trypan blue, sodium bicarbonate, bovine insulin, and dimethyl sulfoxide—were purchased from Sigma-Aldrich Chemical Co. (St. Louis, MO, USA); Dulbecco’s Modified Eagle Medium (DMEM) and trypsin were purchased from Life Technologies Corporation (Carlsbad, CA, USA). The materials used for flow cytometry were purchased from the following companies: rhodamine 123 and propidium iodide were purchased from Thermo Fisher (Waltham, MA, USA); and binding buffer and Annexin-V FITC were purchased from Biosciences (BD, Franklin Lakes, NJ, USA). Murine breast adenocarcinoma cells (4T1) were donated by Professor Dra Suzanne Ostrand-Rosenberg (University of Maryland, Baltimore County, Baltimore, MD, USA).

### 2.2. Development of Pequi Oil Nanoformulations Containing DTX or AA

The development of the nanoemulsions was an adaptation of a method previously reported [21]. First, AA and DTX were dispersed separately in a mixture containing pequi oil and ethanol. The solvent was then removed by rota-evaporation. The films formed by pequi oil and the compounds were resuspended with phosphate buffer containing egg lecithin (pequi oil:egg lecithin at proportion 1:2 (*w*/*w*)) and submitted to sonication at altered pulse of 40% amplitude for 3 min under an ice bath. Then, the nanoformulation was further diluted with phosphate buffer were added to each solution and sonicated a second time at the same conditions previously described. Pequi oil-based nanoemulsion without compound and the blank formulation (without compound and pequi oil) were similarly prepared as described above. The developed formulations were stored at 4 °C and under dark conditions until further analysis, in view of their better stability in this condition as evidenced by previous studies [21].

### 2.3. Dynamic Light Scattering (DLS) and Stability under Time

The nanodroplet hydrodynamic diameter (DH) as well as the polydispersity index (PdI) were determined by the dynamic light scattering (DLS) method. The surface charge was determined by the electrophoretic mobility method. The stability of the formulations stored at 4 °C according to these parameters was evaluated for 60 days. All analyses were performed with the Zetasizer^®^ equipment (DLS, Malvern, Chester County, PA, USA).

### 2.4. Analysis of Infrared Spectrophotometry—FTIR

For the FTIR measurements, the nanoemulsions were previously lyophilized (Thermo Savant SPD121P—Thermo Scientific, Waltham, MA, USA) and stored under nitrogen atmosphere until analysis. The FTIR experiments were performed using a Bruker spectrometer (model Vertex 70). Analysis was performed using the module attenuated total reflectance (ATR, Attenuated Total Reflectance). The measurements were averaged over 96 scans, which were taken at a resolution of 4 cm^−1^ from 400 to 4000 cm^−1^. The background signal was averaged over 96 scans before each measurement. Finally, the spectra were fitted using Gaussian functions. Data were treated with IR Solution software 1.50^®^ and transferred to the graphics program GraphPad Prism 5.0^®^ construction.

### 2.5. Electronic Transmission Microscopy

A morphological analysis of the nanoemulsions was performed by transmission electron microscopy (TEM). The samples were diluted 1:100 (*v*:*v*) in distilled water, and 3 µL of this solution were diluted in copper screens covered with formvar film. The samples were dried at room temperature, and then they were contrasted with osmium tetroxide (OsO_4_) steam for 5 min. The images were acquired using a transmission electron microscope (JEOL JEM-1011, Akishima, Tokyo, Japan) at 80 kV.

### 2.6. Cell Culture

The cell lines murine triple-negative breast cancer (4T1) and murine fibroblast (NIH-3T3) were grown in Dulbecco’s Modified Eagle’s Medium (DMEM) supplemented with 10% of heat-inactivated fetal bovine serum (*v*/*v*) or 10% of calf serum (*v*/*v*), respectively, and 1% of antibiotic solution (100 IU/mL Penicillin—100 µg/mL Streptomycin—*v*/*v*) at 37 °C and 5% CO_2_ in humid atmosphere.

### 2.7. Cell Treatment

For viability assay, cells were seeded into 96-well culture plate at a density of 2 × 10^3^ cells per well (4T1) in DMEM culture medium overnight at 37 °C, 5% CO_2_ in humid atmosphere. Then, the medium was changed, and various concentrations of pequi oil-based nanoemulsion (PeNE), pequi oil-based nanoemulsion associated to AA (PAA), pequi oil-based nanoemulsion associated to DTX (PDTX), blank (BR), free AA, free DTX, and free pequi oil (OL) were added. The concentrations tested were 90–180–360–540 µg/mL given pequi oil, 5–10–20–30 µg/mL of AA, and 8–17–33–50 µg/mL of DTX. The free pequi oil was previously diluted in ethanol. The final ethanol concentration was lower than 1% per well. The plates were incubated for 24 and 48 h at 37 °C, 5% CO_2_ in humid atmosphere.

Combinations of sample were also tested. Cells were seeded into 96-well culture plate at a density of 2 × 10^3^ cells per well (4T1) and 3 × 10^3^ cells per well (NIH-3T3) in DMEM culture medium overnight at 37 °C, 5% CO_2_ in humid atmosphere. The medium was changed, and cells were exposed to “PeNE”, “PAA”, “PDTX”, “AA”, “DTX”, “PAA + PDTX”, and “AA + DTX”. The concentrations tested were 135–180 and 360 µg/mL, given pequi oil, 7–10 µg/mL of AA, and 12–16 µg/mL of DTX. The plates were incubated for 24 and 48 h at 37 °C, 5% CO_2_ in humid atmosphere. Combination index (CI), dose-reduction index (DRI), and fraction affected (Fa) were calculated using CompuSyn^®^ software (Version 1.0) and interpreted according to Chou and Talalay method (1983) [33], given CI < 1, CI = 1, and CI > 1 as synergysm, additive effect, and antagonism, respectively.

### 2.8. Cell Viability Assay

Cell viability assay was performed using MTT (3-[4,5-dimethylthiazol-2-yl]-2,5-diphenyltetrazolium bromide) assay. The MTT assay is based on the reduction of tetrazolium derivatives in living cells by mitochondrial dehydrogenases, allowing the estimation of the metabolic activity of cells. This assay enables the assessment of cell viability and proliferation as parameters of cell survival and growth [34]. After 24 and 48 h of incubation, the treatments were removed, and 150 µL of the MTT solution (0.5 mg/mL in DMEM) was added in each well. The plates were incubated for 2 h at 37 °C and 5% CO_2_ in humid atmosphere. The culture medium was discarded, and 150 µL of dimethyl sulfoxide (DMSO) was added in each well. The absorbance was monitored using a spectrophotometer with a microplate reader at 595 nm (Molecular Devices, EUA). The control group was considered to have 100% cell viability.

### 2.9. Plasma Membrane Integrity and Cell Count

Membrane integrity and cell proliferation evaluation by trypan blue assay was performed in a 24-well plate containing 2 × 10^4^ cells/well. After 24 h of exposure to PeNE, PAA, PDTX, AA, DTX, PAA + PDTX, and AA + DTX at 180 µg/mL—given pequi oil, 10 µg/mLof AA, and 16 µg/mL of DTX—cells were trypsinized, centrifuged, and the pellet obtained was resuspended in 100 μL of culture medium. The cells were stained with a trypan blue solution (0.4% in PBS—Sigma, St. Louis, MO, USA). The number of total cells was determined in Neubauer’s chamber, counting stained or non-stained cells. Cells with intact membranes were considered as non-stained cells and stained blue as cells with damaged plasma membranes. Cells incubated with PBS were used as negative control.

### 2.10. Flow Cytometry

#### 2.10.1. Cells Treatment

For flow cytometry experiments, 4T1 cells were plated into 24-well culture plate at a density of 2 × 10^4^ cells. After incubation for 24 h at 37 °C, 5% CO_2_ in humid atmosphere, the cells were incubated with 0.5 mL of PeNE, PAA, PDTX, AA, DTX, PAA + PDTX, and AA + DTX at 180 µg/mL, given pequi oil, 10 µg/mL of AA, and 16 µg/mL of DTX. Cells incubated with PBS were used as control. After 24 h of treatment, the cells were harvested by trypsinization with 5 min of exposure to trypsin at 37 °C, 5% CO_2_ in humid atmosphere. Then, the trypsin was neutralized with complete culture media, and the cells were centrifuged at 3083× *g* for 5 min at 4 °C in order to be further prepared for the assays described below. Then, each assay was analyzed by flow cytometer (BD FACSVerse^TM^, Piscatway, NJ, USA), and a total of 10,000 events were collected per sample.

#### 2.10.2. Lysosomal Membrane Permeabilization

Acridine orange (AO) is a lysosome-tropic metachromatic fluorochrome. When excited with blue light, AO emits red fluorescence at high concentrations when it is present in lysosomes [35]. Cells were exposed to 50 µM of AO for 30 min and were protected from light at room temperature. After incubation, the lysosomal membrane permeabilization was analyzed by flow cytometry using PI channel.

#### 2.10.3. Mitochondrial Membrane Potential and Cells Morphologic Aspects

The mitochondrial membrane potential analysis was performed to evaluate potential mechanisms associated with apoptosis, using Rhodamine 123, a cationic fluorescent probe able to accumulate specifically in the mitochondria due to the negative transmembrane potential in this organelle in living cells. Cells were washed twice in PBS, and then 300 µL of Rhodamine 123 (5 µg/mL in PBS) was added to each sample and incubated for 15 min at room temperature, protected from light. Then, the cells were washed twice with PBS and were analyzed. Parameters related to the size and granularity of treated cells were obtained by flow cytometry using FSC and SSC channels, respectively.

#### 2.10.4. ROS Level

Reactive Oxygen Species (ROS) level was analyzed using H_2_DCFDA (5-(and-6)-chloromethyl-29, 79-dichlorodihydrofluoresceindiacetate, acethyl ester). Unspecific esterase cleaved H_2_DCFDA in DCFDA that turns into fluorescent molecule of DCF with the presence of intracellular ROS [36]. The cells were exposed to 100 µL of H_2_DCFDA at 10 µM final concentration for 40–60 min, protected from light at room temperature. ROS level was then analyzed by flow cytometry using FITC channel.

#### 2.10.5. DNA Fragmentation Assay and Cell-Cycle

The cells were resuspended in 1 mL of cold ethanol (70%) and stored at −30 °C for 24 h. After the incubation time, the cells were washed twice in PBS, and 100 µL of RNAse (50 µg/mL) was added for 30 min at 37 °C, protected from light. Then, 100 µL of propidium iodide (PI—20 µg/mL in PBS) was incubated for 30 min at room temperature, protected from light. The DNA fragmentation and cell cycle were analyzed by flow cytometry using PI channel.

#### 2.10.6. Annexin-V FITC/Propidium Iodide (PI) Staining

The annexin-V FITC/PI staining was applied to distinguish apoptotic cells and necrotic cells. The cells were washed in PBS and resuspended in 100 µL of binding buffer [10 mM HEPES/NaOH (pH 7.4), 140 mM NaCl, 2.5 mM CaCl_2_]. Then, 5 µL of Annexin-V FITC was added and incubated for 15 min in the dark at room temperature. Next, 10 µL of PI (50 µg/mL) and 200 µL of binding buffer were added, and the sample was analyzed by flow cytometry using FITC and PerCP channels.

#### 2.10.7. Multicaspase Assay

Multicaspase assay was perfomed using Muse^®^ MultiCaspase Kit (Merck, Darmstadt, Germany). Basically, cells were resuspended in caspase buffer 1×, and 5 µL of Muse^®^ Multicaspase working solution was added to each sample. After 30 min of incubation at 37 °C, protected from light, 150 µL of 7-AAD working solution was added. The samples were analyzed by flow cytometer (Muse^®^ Cell Analyzer, Mannheim, Germany). A total of 5000 events were collected per sample.

### 2.11. Clonogenic Assay

Clonogenic assay was performed to measure the ability of cells to form colony. Cells were seeded at density of 2 × 10^4^ cells per well in 24-well culture plate. After 24 h at 37 °C, 5% CO_2_ in humid atmosphere, the cells were exposed to PeNE, PAA, PDTX, AA, DTX, PAA + PDTX, and AA + DTX at 180 µg/mL—given pequi oil, 10 µg/mLof AA and 16 µg/mL of DTX—for 24 h. After time incubation, cells were plated in 6-well culture plates at the density of 1000 cells per well in complete media and cultured for another 5 days at 37 °C, 5% CO_2_ in humid atmosphere without changing medium. Then, the media were removed, colonies were washed twice with cold PBS, fixed with methyl alcohol for 5 min at room temperature, and stained with 0.5% violet crystal dye in 25% methyl alcohol and 75% distilled water for 10 min at room temperature. Cells were then washed with distilled water, and the number of colonies containing at least 50 cells was determined. The surviving fractions were calculated using Equations (1) and (2):

(1) $$Plating\;efficiency\;\left(PE\right)=\frac{\#\;colonies\;formed}{\#\;of\;cells\;seeded}\times 100\%$$

(2) $$Survival\;fraction\;\left(FS\right)=\frac{\#\;of\;colonies\;formed\;after\;treatment}{\#\;of\;cells\;seeded\times PE}$$

### 2.12. Cell Morphology Analysis by Optical Microscopy

The 4T1 cell morphology was evaluated by optical microscopy images after 24 h of treatment with PeNE, PAA, PDTX, AA, DTX, PAA + PDTX, and AA + DTX at 180 µg/mL—given pequi oil, 10 µg/mL of AA and 16 µg/mL of DTX—in cells seeded in 12-wells plates (3 × 10^4^ cells/well). Cells incubated with PBS were used as a negative control. Each treatment was performed in triplicate. After 24 h, wells were analyzed by Leica DMi1 microscope with 20× objectives. The images were captured by Leica MC170 HD camera.

### 2.13. Statistical Analysis

Statistical differences between control and treated cells were evaluated by the analysis of variance (ANOVA) and Tukey post hoc test at a significance level of 0.05 using Graph Pad Prism 6.02 (GraphPad Software, La Jolla, CA, USA) with Shapiro–Wilk. All data presented normal distribution, and values were expressed as means ± standard error of the mean (SEM), and a value of *p* < 0.05 was considered statistically significant. All assays were performed in triplicates in three independent experiments.

## 3. Results and Discussion

### 3.1. Preparation and Characterization of Nanoemulsions

Nanoemulsions have been increasingly used as delivery systems for hydrophobic drugs in cancer therapies, including breast cancer [15,18,37]. Herein, we investigated for the first time the cytotoxicity of the combination approach in incubating PeNE associated with AA and DTX in triple-negative breast cancer cells in vitro.

Firstly, the nanoformulations were obtained, and their physicochemical properties were evaluated. Studies have reported that the mean hydrodynamic diameter (HD) of nanoemulsion droplets should be less than 500 nm [15,18]. However, some other studies considered a mean size below 200 nm [38]. The nanoformulations obtained showed HD < 200 nm and PdI < 0.3 (Table 1), indicating a homogeneous size distribution [39,40]. All formulations presented a negative zeta potential of approximately –20 mV at neutral pH (Table 1).

**Figure 1:**
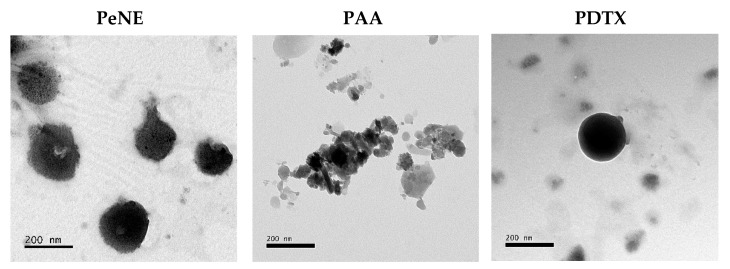
Shape of pequi oil nanoemulsion (PeNE), pequi oil nanoemulsion associated with anacardic acid (PAA), and docetaxel (PDTX), evaluated by transmission electron microscopy. Scale bar = 200 nm.

Moreover, electronic transmission microscopy analysis showed that PeNE, PAA, and PDTX presented a spherical shape (Figure 1). Yadav&Gupta (2013) also reported the spherical shape of a DTX-loaded nanoemulsion stabilized by soy phosphatidylcholine [41]. Similarly, AA-containing liposomes showed circular structures by TEM analysis [42].

**Figure 2:**
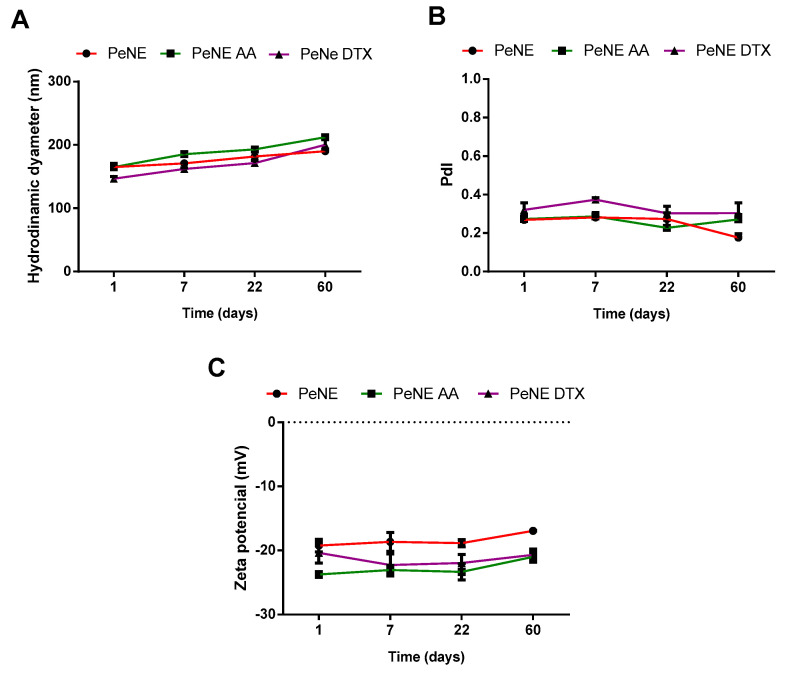
Stability of pequi oil of pequi oil nanoemulsion (PeNE), pequi oil nanoemulsion associated to anacardic acid (PAA), and docetaxel (PDTX) at 4 °C storage according to hydrodynamic diameter (**A**), polydispersity index (**B**), and zeta potential (**C**). One-way ANOVA *p* < 0.05 (Tukey post hoc test).

The stability of nanoemulsions is an important parameter to be evaluated, particularly when considering their future biomedical applications. Nanoemulsions are kinetically stable but thermodynamically unstable. Consequently, flocculation, coalescence, gravitational separation, and Ostwald ripening are examples of some instability that can be observed [43,44]. In the present study, all nanoemulsions remained stable at 4 °C storage temperature during the period evaluated (60 days) (Figure 2), corroborating with previous studies of our group [21].

**Figure 3:**
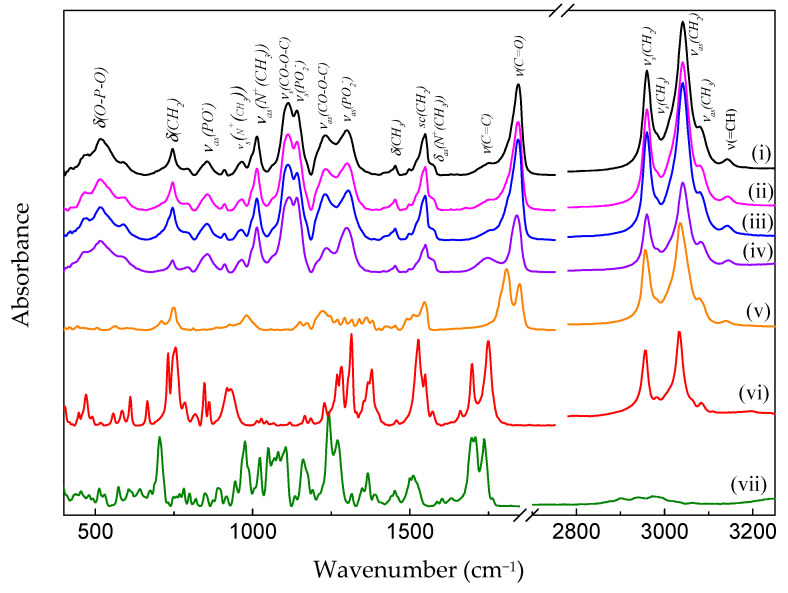
FTIR spectrum of pequi oil nanoemulsion (PeNE) (i), pequi oil nanoemulsion associated to anacardic acid (PAA) (ii), pequi oil nanoemulsion associated to docetaxel (PDTX) (iii), blank formulation (only lecithin) (iv), pure pequi oil (v), free anacardic acid (vi), and free docetaxel (vii).

Figure 3 shows the FTIR spectra of the nanoemulsions PeNE (i), PAA (ii), PDTX (iii), lecithin bilayer (iv), pure pequi oil (v), free AA (vi), and free DTX (vii). It can be observed that the spectra of the PeNE, PAA, and PDTX present as spectral characteristics of the lecithin bilayer, with bands associated with the polar head at 970 and 1234 cm^−1^, referring to the vibration modes $\nu_{as}(N^{+}{\left(CH_{3}\right)}_{3})$ and $\nu_{as}\left({PO}_{2}^{-}\right)$. The absorption bands of the methylene and methyl groups from the hydrocarbon chain are found at 721 ($\delta (CH_{2}))$, 1380 ($\delta (CH_{3}))$, 1465 ($\tau (CH_{2}))$, 2852 ($\nu_{s}({CH}_{2})$), 2920 ($\nu_{as}({CH}_{2})$) and 2955 cm^−1^ ($\nu_{as}({CH}_{3})$) [45].

Since pequi oil and lecithin show very similar molecular composition, with the exception of the vibrations from the polar head group of the phosphatidylcholines that make up lecithin, it is not possible to distinguish the individual contributions of each component in the FTIR spectra (see Figure 3, spectra (iv) and (v)). Furthermore, by comparing the FTIR spectra of the nanoemulsions (Figure 3 (i–iv)) with the spectra of the AA (Figure 3 (vi)) and DTX (Figure 3 (vii)), one can observe that there is obvious spectral evidence of the presence of the latter in the spectra of nanoemulsions. Therefore, the following discussion refers to the vibrational modes of the hydrocarbon chains from both molecular components that make up the nanoemulsions. At last, the stretching vibrations of the carbonyl ester group ν (C=O) are found to be around 1740 cm^−1^.

**Figure 4:**
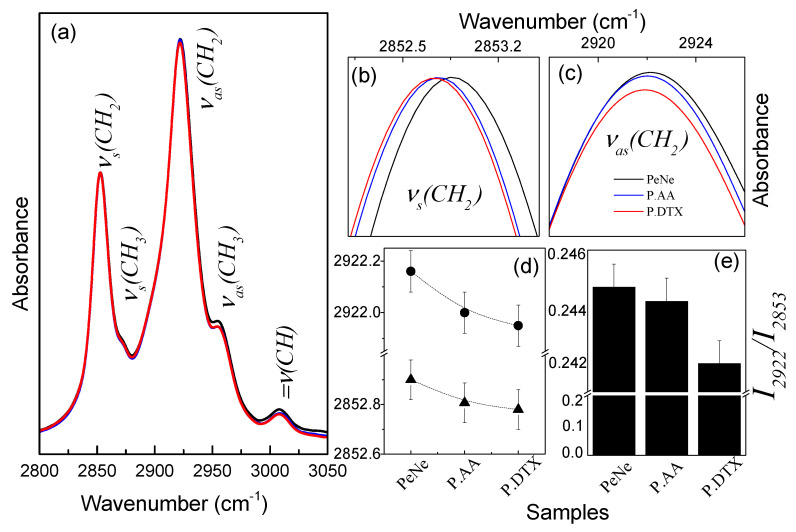
(**a**) FTIR spectrum in the region of 2800 to 3050 cm^−1^ of the pequi oil nanoemulsion (PENE—black line), pequi oil nanoemulsion associated to anacardic acid (PAA—blue line), and pequi oil nanoemulsion associated to docetaxel (PDTX—red line). Magnification of spectral regions around 2852 cm^−1^ (**b**) and 2922 cm^−1^ (**c**). Dependence on vibrational energies (**d**) and the ratios between the intensities of the absorption bands $\nu_{s}({CH}_{2})$ and $\nu_{as}({CH}_{2})$ as a function of the different nanoemulsions (**e**).

The assumption that the drug impacts the arrangement of the hydrocarbon chains is supported by the absorption bands of the methylene group’s stretching modes ($\nu ({CH}_{2}$) (see Figure 4). Note that, although small, the vibrational energies, as well as the ratios between the intensities ($I_{\nu_{as}({CH}_{2})}/I_{\nu_{s}({CH}_{2})}$) of the symmetric and asymmetric stretching modes of the group ${CH}_{2}$ decrease in the same sequence as before: PeNE, PAA, and PDTX (Figure 4b,e).

It is well-established that the stretching vibrations of the ${CH}_{2}$ group are influenced by the conformation of the hydrocarbon chain and are responsive to changes in the proportion of trans/gauche rotamers of the fatty acid acyl chains [46]. For instance, the conformational disorder of the all-trans acyl chain is accompanied by an increase in vibrational energies and an increase in the ratio between the intensities $I_{2922}/I_{2853}$ of the bands associated with the ${CH}_{2}$ group of lipid hydrocarbon chains [47].

Based on the previous discussions, it can be proposed that incorporating DTX or AA into pequi oil nanoemulsion induces a conformational ordering in the hydrocarbon chains within the nanoemulsions. DTX promotes a higher ordering than AA, which in turn is greater than the nanoemulsion without the compounds (Figure 4).

### 3.2. Combined Effect of PDTX and PAA on Triple Negative Breast Cancer Cells (4T1)

Cell viability was analyzed after 24 and 48 h of exposure by MTT assay (Table 2). It was observed that PeNE significantly decreases cell viability at 180 µg/mL (20 and 30% after 24 and 48 h, respectively, *p* < 0.0001), corroborating our previous studies demonstrating dose- and time-dependent cytotoxicity of PeNE in 4T1 cells [21,22,31]. The encapsulation of DTX and AA in PeNE nanodroplets enhanced PeNE cytotoxicity. Specifically, a decrease in cell viability by 34% and 50% (*p* < 0.0001) was observed after 24 h at a concentration of 180 µg/mL (corresponding to pequi oil). After 48 h, this reduction was higher, with cell viability decrease of 60 and 64% (*p* < 0.0001), respectively. Moreover, it was noted that the encapsulation of AA and DTX improved their cytotoxic activity compared to their free form. AA did not induce cytotoxicity in tested concentrations (7 and 10 µg/mL), and DTX only reduced approximately 30% of cell viability at both concentrations 12 and 16 µg/mL (*p* < 0.0001) after 24 and 48 h. Unexpectedly, the combination of DTX + AA did not enhance the cytotoxic effect of either molecule. Instead, free AA + DTX demonstrated cell viability reduction similar to free DTX. However, and interestingly, PAA + PDTX showed improved cytotoxic activity when compared to all other groups, including PAA and PDTX alone, at higher concentrations (180 µg/mL of pequi oil, 10 µg/mL of AA + 180 µg/mL of pequi oil, and 16 µg/mL of DTX). PAA + PDTX induced a reduction in cell viability by approximately 70 and 90% (*p* < 0.0001) after 24 and 48 h, respectively.

Combination index values were calculated to assess whether the association of AA and DTX in PeNE and the combination of AA + DTX and PAA + PDTX resulted in synergism, additive effect, or antagonism (Table 3). Chou and Talalay (1983) elaborated a combination index (CI) theorem that defines additive effect (CI = 1), synergism (CI < 1), and antagonism (CI > 1) in drug combinations [33]. Herein, we demonstrated that AA + DTX induced antagonism (CI = 2.572). As observed previously in the MTT assay, the presence of AA did not impact the cytotoxicity of DTX. However, the association of AA and DTX in PeNE led to synergism (CI = 0.913 and 0.640, respectively). Additionally, the association of AA and DTX encapsulated (PAA + PDTX) demonstrated an additive effect (CI = 0.991), suggesting that the use of nanotechnology enhances cytotoxic outcomes.

The use of combination therapy enhanced the improved cytotoxic response rates compared to treatments using a single chemotherapeutic drug [48,49].

In the present work, we proposed that the combination of bioactive compounds present in pequi oil along with AA and DTX may have improved the tumor cells’ sensitivity to the treatment.

For the next assays, the concentrations of 180 µg/mL of pequi oil and 10 µg/mL of AA and/or 180 µg/mL of pequi oil and 16 µg/mL of DTX were chosen to investigate their effects on cell proliferation and key organelles.

### 3.3. PDTX + PAA Modified the Morphology of Breast Cancer Cells

**Figure 5:**
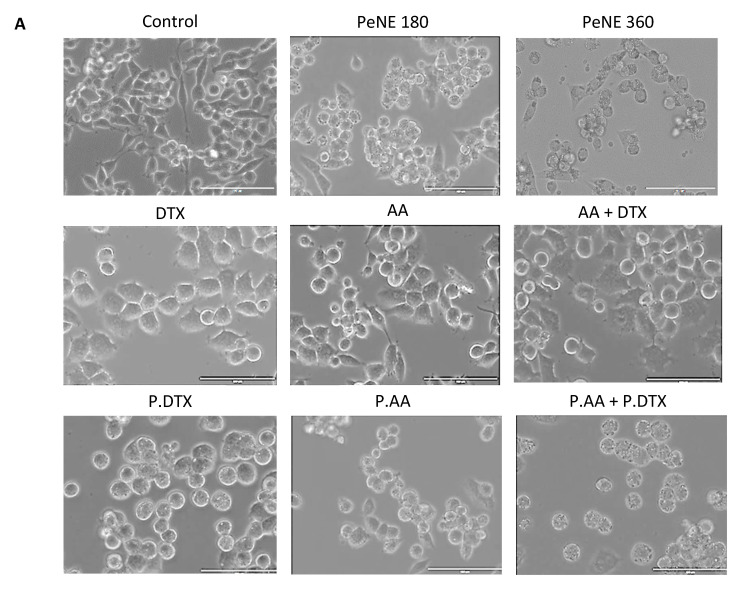

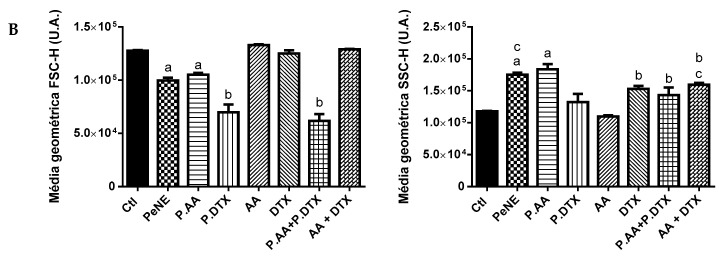
Morphologic evaluation of triple-negative breast cancer cells (4T1) by contrast phase microscopy (**A**) and flow cytometry (**B**) after 24 h of exposure with pequi oil nanoemulsion (PeNE), pequi oil nanoemulsion associated with anacardic acid (PAA), and docetaxel (PDTX), free anarcadic acid (AA), free docetaxel (DTX), association of PAA and PDTX (P.AA + P.DTX), and association of free AA and free DTX (AA + DTX) at 180 µg/mL of pequi oil, 10 µg/mL of AA, and 16 µg/mL of DTX. Control group was treated with phosphate buffer. (**A**) Scale bar = 100 µm. (**B**) FSC-H = size and SSC-H = granularity. One-way ANOVA: significant difference between groups *p* < 0.05 (Tukey post hoc test). Different letters indicate statistically significant differences between groups.

Changes in cell morphology suggest alterations in physiological processes and potential cell death. The morphology of 4T1 cells exposed to the nanoformulations for 24 h was evaluated by contrast phase microscopy and flow cytometry (Figure 5). Cells in the control group exhibited the typical 4T1 morphology, characterized by a mix of rounded and elongated cells (Figure 5A). No significant changes were observed in cells exposed to AA alone. However, cells exposed to all other treatments (DTX, AA + DTX, PeNE, PAA, PDTX, and PAA + PDTX) exhibited morphological changes, including a higher density of rounded cells and the presence of vacuoles (Figure 5A).

Analysis of size (FSC-H) and granularity (SSC-H) was consistent with the microscopic observations. Cells treated with AA did not undergo expressive morphological changes. However, a significant reduction in cell size was observed in those exposed to PeNE, PAA, PDTX, and PAA + PDTX (22–18–46, and 52%, respectively, *p* < 0.0001). Regarding granularity, only cells exposed to PDTX and AA did not present significant changes when compared to the control group.

Various morphological changes are linked to cell death mechanisms such as apoptosis, necrosis, and autophagy. Cell shrinkage and the formation of vacuoles (apoptotic bodies) are recognized as characteristic features of apoptosis. However, additional analysis is required to confirm the specific cell death mechanism.

### 3.4. PAA + PDTX Significantly Impacted 4T1 Key Organelles

**Figure 6:**
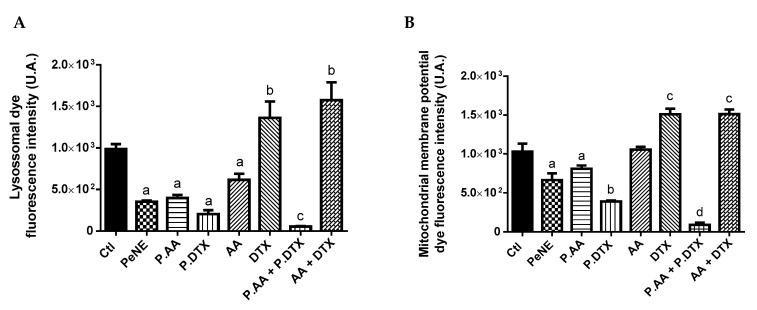

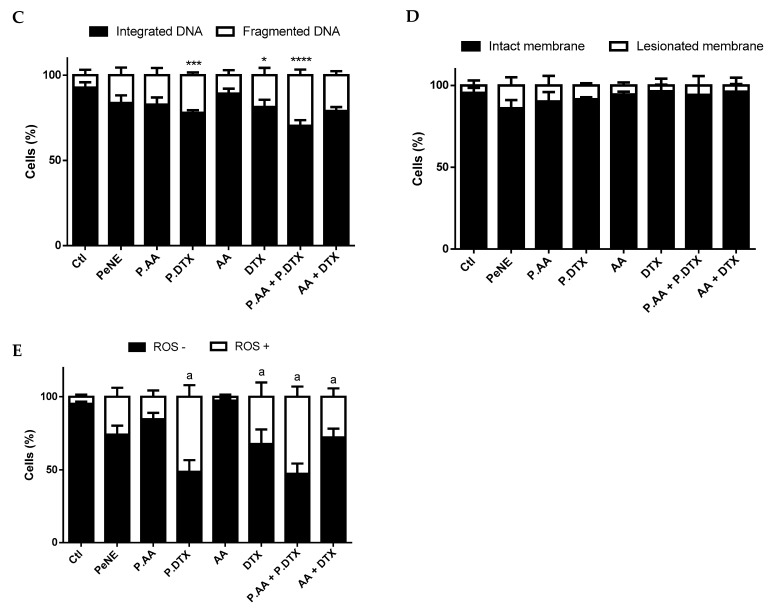
Assessment of cytotoxic effect on key organelles, plasma membrane, and intracellular physiology of triple-negative breast cancer cells (4T1) by flow cytometry. The cells were exposed to pequi oil nanoemulsion (PeNE), pequi oil nanoemulsion associated with anacardic acid (P.AA), and docetaxel (P.DTX), free anarcadic acid (AA), free docetaxel (DTX), association of PAA e PDTX, and association of free AA and free DTX (AA + DTX) at 180 µg/mL of pequi oil, 10 µg/mL of AA and 16 µg/mL of DTX for 24 h. (**A**) Lysosomal membrane permeability. (**B**) Mitochondrial membrane potential. (**C**) Fragmentation of DNA. (**D**) Membrane integrity by trypan blue assay. (**E**) Intracellular Reactive Oxygen Species (ROS) analysis with CM-H_2_DCFDA. One-way and two-way ANOVA: significant difference between groups with *: *p* < 0.05, ***: *p* < 0.001 and ****: *p* < 0.0001 (Tukey post hoc test). Different letters indicate statistically significant differences between groups.

Additional analysis was performed to investigate the effect of samples in different key organelles, plasma membranes, and in some physiological processes, like intracellular ROS production, of 4T1 cells (Figure 6).

The lysosome is an acidic organelle containing numerous hydrolase enzymes responsible for recycling cellular components. Recently, it has also been found to play a role in regulating cell metabolism and growth. Lysosomal function is known to contribute to carcinogenesis, making it a promising target for cancer treatment [50,51]. After 24 h of exposure, we measured the lysosomal membrane permeability, which was analyzed by flow cytometry using acridine orange (AO), a specific dye for acidic organelle. A significant reduction of AO accumulation into lysosome was observed after exposure to PeNE, PAA, PDTX, AA, and PAA + PDTX (*p* < 0.0001) (Figure 6A). PAA + PDTX showed a more expressive decrease compared to other groups. This reduction suggests the release of lysosomal content into cytosol and a decrease of its acidity environment. DTX and AA + DTX significantly enhanced the accumulation of AO in the lysosome. Zhang and coworkers (2018) also reported an increase in lysosomal acidification after DTX exposure to gastric cells in vitro [52]. Exposure to certain drugs can induce lysosomal biogenesis, leading to an increased number of lysosomes per cell, an expansion in lysosomal volume, and heightened enzyme activity. As a result, hydrophobic drugs may become sequestered within the lysosomal compartment, limiting their access to target sites and diminishing their cytotoxic effect [52,53]. In this study, DTX and AA + DTX result in higher acidity of the lysosome when compared to the control group and also when compared to PDTX and PAA + PDTX, respectively. Moreover, PDTX and PAA + PDTX also demonstrated higher cytotoxic activity than DTX and AA + DTX (Table 2), indicating that the use of nanotechnology might impact the interactions and intracellular targets of the drugs.

Lately, lysosomal membrane permeabilization has been recognized as an initiating step in the cell death process, leading to subsequent alterations in other organelles, such as mitochondria. Cell exposure to PeNE, PAA, PDTX, and PAA + PDTX led to depolarization of the mitochondrial membrane (*p* < 0.0001) (Figure 6B). Depolarization was at least 30% higher after exposure to PAA + PDTX when compared to related groups and 90% higher when compared to the control group. In parallel, DTX and AA + DTX induced an increase in fluorescence intensity, suggesting hyperpolarization (*p* < 0.0001).

In addition, the impact of the treatments on DNA integrity was investigated. DNA fragmentation is another remarkable signal of the cell death mechanism, primarily associated to apoptosis. A significant increase in DNA fragmentation was observed after exposure to DTX (*p* < 0.05), PDTX (*p* < 0.001), and PAA + PDTX (*p* < 0.0001) when compared to the control group (Figure 6C).

Additionally, the plasma membrane remained intact after exposure to all samples (Figure 6D). Nevertheless, a tendency of PeNE to increase (~10%) damage to the plasma membrane was observed when compared to the control group. This result is consistent with data reported in our previous study [22].

All samples containing DTX (DTX, PDTX, AA + DTX, and PAA + PDTX) induced significant intracellular ROS production (*p* < 0.0001) (Figure 6E). Elevated levels of intracellular ROS are implicated in the disruption of organelle functions, including those of mitochondria and lysosomes [35,54]. Therefore, the effects observed on these organelles herein might be linked to these mechanisms.

### 3.5. PAA + PDTX Reduced Cell Proliferation

**Figure 7:**
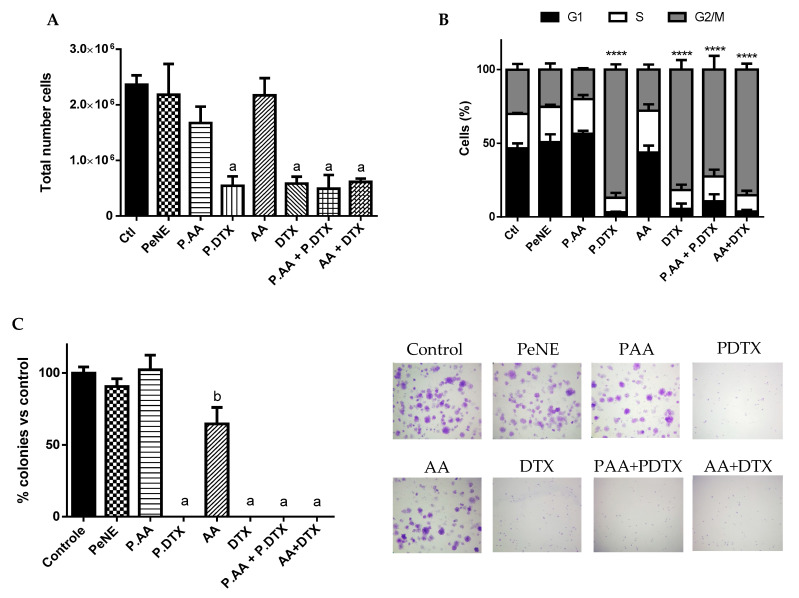
Impact on cell proliferation. The cells were exposed to pequi oil nanoemulsion (PeNE), pequi oil nanoemulsion associated with anacardic acid (PAA) and docetaxel (PDTX), free anacardic acid (AA), free docetaxel (DTX), association of PAA + P. DTX, and association of free AA and free DTX (AA + DTX) at 180 µg/mL of pequi oil, 10 µg/mL of AA and 16 µg/mL of DTX for 24 h. (**A**) Total cell number by trypan blue assay. (**B**) Cell cycle using propidium iodide by flow cytometry. (**C**) Clonogenic assay for formation of colonies. (**A**,**C**) One-way ANOVA: significant difference between groups *p* < 0.05 (Tukey post hoc test). Different letters indicate statistically significant differences between groups. (**B**) Two-way ANOVA: significant difference between groups **** *p* < 0.001 (Tukey post hoc test).

All samples containing DTX (DTX, PDTX, AA + DTX, and PAA + PDTX) significantly decreased the total number of cells (~77%), blocked the cell cycle in the G2/M phase (~80%), and induced a 100% inhibition on the capacity of cancer cells to form colonies (*p* < 0.0001) (Figure 7). These results showed an inhibition of cell proliferation mostly due to the presence of DTX, with no significant difference among groups. DTX is a microtubule-stabilizing antitumor drug that can inhibit cell mitosis and reduce cell proliferation, ultimately leading to cell death [55]. Maroufi and coworkers (2020) reported sub-G1 cell cycle arrest of MDA-MBA231 breast cancer cells in vitro after treatment with DTX at 5 nM for 48 h [56].

In parallel, AA demonstrated a reduction of cell capacity to form colonies (~40%, *p* < 0.05) but did not reduce total cell number and did not alter cell cycle (Figure 7). In corroboration with our results, AA reduced cell capacity to form colonies at 5–20 µM in breast cancer cells MDA-MB-231 in vitro. However, the same study also reported that AA inhibited cell proliferation and induced G0-G1 cell cycle arrest at 25–100 µM in a dose-dependent way (higher concentration than our study) [11].

### 3.6. PAA + PDTX Promoted Phosphatidylserine Exposure and Multicaspase Activation

**Figure 8:**
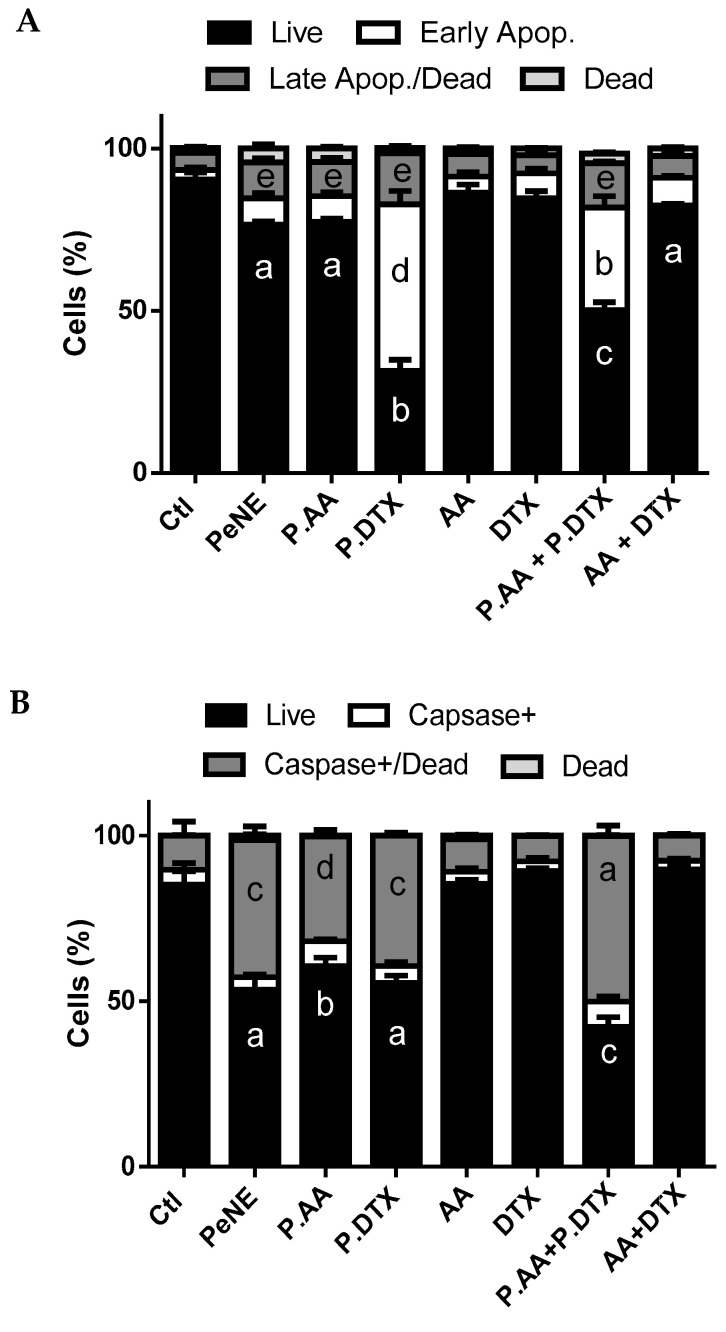
Exposition of phosphatidylserine and caspase activation. The cells were exposed to pequi oil nanoemulsion (PeNE), pequi oil nanoemulsion associated with anacardic acid (PAA) and docetaxel (PDTX), free anarcadic acid (AA), free docetaxel (DTX), association of PAA + PDTX and association of free AA and free DTX (AA + DTX) at 180 µg/mL of pequi oil, 10 µg/mL of AA, and 16 µg/mL of DTX for 24 h. (**A**) Exposition of phosphatidylserine (stained with annexin V-FITC and propidium iodide by flow cytometry). (**B**) Multicaspase activity. One-way ANOVA: significant difference between groups *p* < 0.05 (Tukey post hoc test). Different letters indicate statistically significant differences between groups.

In the present work, early apoptosis was observed in cells exposed to PDTX and PAA + PDTX (50 and 30%, respectively) (*p* < 0.0001). A small increase of late apoptosis/dead (~5%) was observed (*p* < 0.05) in cells exposed to PeNE, PAA, PDTX, and PAA + PDTX, and no change in dead cell proportion was noted compared to the control group (Figure 8A).

Moreover, PeNE, PAA, PDTX, and PAA + PDTX promoted caspase activation with significant improvement of caspase^+^/dead cells (*p* < 0.0001) (Figure 8B). PAA + PDTX resulted in greater caspase activation compared to the other groups (*p* < 0.0001).

The exposure of phosphatidylserine and the activation of caspase are indicators of apoptosis. Both were observed in cells treated with PDTX and PAA + PDTX. Furthermore, there was no change in the proportion of dead cells, indicating no plasma membrane damage and, consequently, no cell death by necrosis.

Caspases play a crucial role in initiating and executing apoptosis through two pathways: one via death receptor engagement (extrinsic) and the other through intracellular stress and the loss of mitochondrial integrity (intrinsic). Previous studies have shown that AA induces apoptosis in 4T1 cells via the extrinsic pathway by activating caspase-8 [57]. Other studies showed that free DTX and DTX nanoparticles activate caspase-3, which participates in apoptosis in both extrinsic and intrinsic pathways [58]. In addition, our data show that PAA + PDTX causes mitochondrial damage and triggers multicaspase activation, indicating that both the extrinsic and intrinsic pathways are activated.

**Figure 9:**
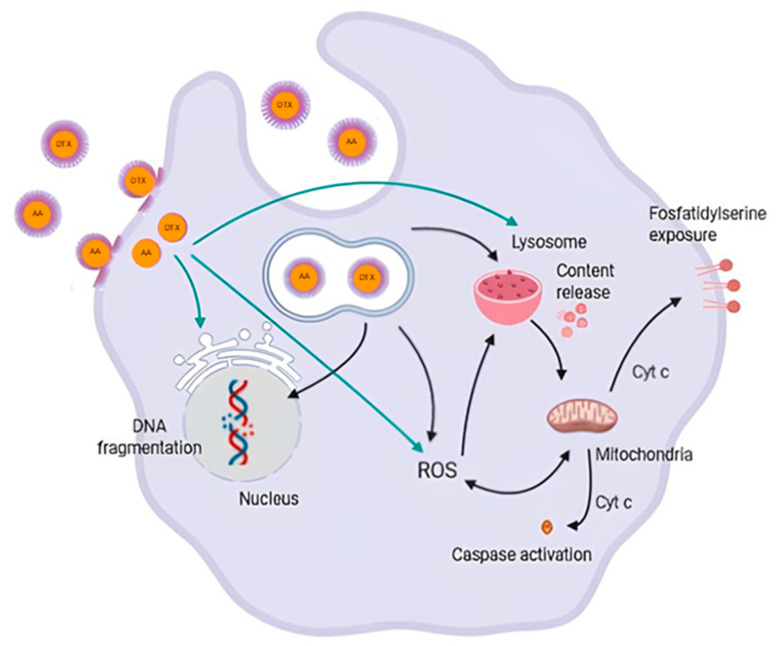
Suggested cell targets involved in the cytotoxicity of pequi oil nanoemulsion associated to docetaxel (PDTX) and anacardic acid (PAA) on breast cancer cells (4T1). Source: own authorship created in BioRender.com.

Taken together, these results suggest that PAA + PDTX was the most promising treatment against 4T1 cells herein, showing a combination effect among the antitumor compounds (Figure 9). The use of nanoemulsions significantly enhanced the anticancer activity when compared to AA + DTX and its antagonism effect. PAA + PDTX affected lysosomal membrane permeability, which may lead to mitochondrial membrane depolarization and, in turn, lead to exposure of phosphatidylserine and activation of caspases. In parallel, alterations in mitochondrial membrane potential may increase ROS production, potentially leading to DNA fragmentation, cell cycle arrest, and, consequently, reduced cell proliferation and changes in cell morphology.

## 4. Conclusions

Natural products are a rich source of bioactive molecules with anticancer properties. The use of pequi oil nanoemulsion (PeNE) as a drug delivery platform has shown to be effective against 4T1 triple-negative breast cancer cells in vitro. Moreover, the association of docetaxel (DTX) and anacardic acid (AA) in PeNE demonstrated to be a promising approach, resulting in combined cytotoxic effects and highlighting the potential of this strategy for breast cancer treatment. This study is the first to report combinatory therapy between pequi oil, DTX, and AA. Further evaluations to verify the efficacy of this treatment on in vivo models are underway.

## Figures and Tables

**Table 1:** Physical and chemical properties of pequi oil nanoemulsion (PeNE), pequi oil-based nanoemulsion associated with anacardic acid (PAA), and docetaxel (PDTX).

	HD (nm)	PdI	Zeta Potential (mv)	pH
PeNE	164.8 ± 6.1	0.269 ± 0.015	−19.2 ± 0.9	7
PAA	164.8 ± 0.7	0.272 ± 0.019	−23.7 ± 0.4	7
PDTX	146.6 ± 3.4	0.257 ± 0.030	−20.3 ± 1.6	7

HD: hydrodynamic diameter; PdI: polydispersity index.

**Table 2:** Cytotoxic impact of pequi oil nanoemulsion (PeNE), pequi oil nanoemulsion associated to anacardic acid (PAA), pequi oil nanoemulsion associated to docetaxel (PDTX) in triple-negative breast cancer cells (4T1) and fibroblasts (NIH3T3) after 24 and 48 h.

				4T1		NIH3T3	
	[Pequi Oil] µg/mL	[AA] µg/mL	[DTX] µg/mL	24 h	48 h	24 h	48 h
Control	-	-	-	100.0 ± 5.2	100.0 ± 19.5	100.0 ± 7.5	100.0 ± 12.6
PeNE	135	-	-	90.2 ± 12.8	91.9 ± 17.1	97.4 ± 7.2	105.8 ± 15.1
	180	-	-	75.1 ± 10.1^a^	68.7 ± 10.5 ^a^	99.1 ± 10.0	77.7 ± 9.4
PAA	135	7	-	79.4 ± 9.3 ^a,b^	62.4 ± 12.2 ^a^	97.8 ± 8.3	97.4 ± 9.4
	180	10	-	66.8 ± 5.9 ^b,d^	40.0 ± 8.3 ^b^	103.5 ± 13.8	158.2 ± 21.3 ^a^
PDTX	135	-	12	57.1 ± 9.6 ^c,d^	40.7 ± 6.2 ^b^	73.6 ± 9.9 ^a^	28.7 ± 5.5 ^b^
	180	-	16	50.7 ± 6.0 ^c,d^	36.8 ± 5.4 ^b^	67.7 ± 13.0 ^a^	16.6 ± 5.6 ^b^
AA	-	7	-	100.5 ± 11.4	115.7 ± 21.2	100.4 ± 11.5	80.4 ± 18.7
	-	10	-	107.7 ± 7.2	187.5 ± 23.0 ^d^	94.5 ± 10.5	84.9 ± 16.4
DTX	-	-	12	74.2 ± 6.0 ^a,b^	73.6 ± 6.8 ^a^	68.1 ± 6.9 ^a^	32.3 ± 5.1 ^b^
	-	-	16	72.9 ± 6.8 ^a,b^	69.0 ± 10.3 ^a^	64.3 ± 8.2 ^a^	42.9 ± 14.2 ^b^
PAA + PDTX	135	7	12	45.8 ± 6.6 ^c^	30.3 ± 8.1 ^b,c^	66.5 ± 4.8 ^a^	18.7 ± 4.2 ^b^
	135	7	12	39.3 ± 6.6 ^c^	25.9 ± 5.7 ^b,c^	59.2 ± 15.9 ^a^	14.6 ± 2.8 ^b^
	180	10	16	40.2 ± 5.9 ^c,e^	17.9 ± 3.8 ^c^	75.8 ± 18.9 ^a^	16.7 ± 3.1 ^b^
	180	10	16	31.4 ± 6.0 ^e^	12.7 ± 2.6 ^c^	41.7 ± 6.9 ^b^	14.4 ± 3.5 ^b^
AA + DTX	-	7	12	76.6 ± 6.8 ^a,b^	81.8 ± 8.7	57.5 ± 7.5 ^a^	45.3 ± 23.1 ^b,c^
	-	7	12	69.8 ± 10.3 ^b^	72.1 ± 8.0 ^a^	59.3 ± 6.8 ^a^	59.4 ± 24.2 ^c^
	-	10	16	76.3 ± 7.3 ^a,b^	77.7 ± 9.4 ^a^	59.0 ± 13.4 ^a^	42.9 ± 21.7 ^b,c^
	-	10	16	65.4 ± 8.9 ^b^	61.0 ± 4.3 ^a^	54.8 ± 4.7 ^a^	48.5 ± 20.0 ^b,c^

The values are expressed as mean ± SEM. One-way ANOVA: significant difference between groups *p* < 0.05 (Tukey post hoc test). Different letters indicate statistically significant differences between groups.

**Table 3:** Combination index values of pequi oil nanoemulsion (PeNE), pequi oil nanoemulsion associated to anacardic acid (PAA), pequi oil nanoemulsion associated to docetaxel (PDTX), free anacardic acid (AA), and free docetaxel (DTX) in triple-negative breast cancer cells (4T1) after 24 h.

	Fa *	DRI ** PeNE	DRI AA	DRI DTX	DRI PAA	DRI PDTX	CI *** Values	Effect
P.AA	0.52	1.82	2.71	-	-	-	0.91361	Synergism
PDTX	0.67	2.62	-	3.84	-	-	0.64067	Synergism
PAA + PDTX	0.68	-	-	-	2.13	1.90	0.99141	Additive
AA + DTX	0.34	-	1.17	0.73	-	-	2.57225	Antagonism

* Fa: Fraction affected (death cells); ** DRI: Dose Reduction Index; *** CI: Combination Index. Considering 180 µg/mL of pequi oil for each pequi oil nanoemulsion and corresponding AA and DTX concentration (10 and 16 µg/mL, respectively).

## Data Availability

The original contributions presented in the study are included in the article, further inquiries can be directed to the corresponding author.
